# Comparing two strategies to support the implementation of evidence-based practices for substance use disorders in VA’s permanent supportive housing program: a protocol for a type 3 hybrid cluster-randomized controlled trial

**DOI:** 10.1186/s13012-026-01498-z

**Published:** 2026-03-24

**Authors:** Sonya Gabrielian, Evelyn T Chang, Emily B. H. Treichler, Jenny Barnard, Richard E. Nelson, Michelle S Wong, Nicholas J. Jackson, Erin P. Finley

**Affiliations:** 1 Department of Psychiatry and Biobehavioral Sciences, David Geffen School of Medicine, University of California, Los Angeles, Los Angeles, CA USA; 2 Center for the Study of Healthcare Innovation, Implementation & Policy (CSHIIP), VA Greater Los Angeles Healthcare System, Los Angeles, CA USA; 3 Division of General Internal Medicine and Health Services Research, Department of Medicine, David Geffen School of Medicine, University of California, Los Angeles, Los Angeles, CA USA; 4 Veterans Affairs San Diego Healthcare System, San Diego, CA USA; 5 Department of Psychiatry, University of California, San Diego, San Diego, CA USA; 6 IDEAS (Informatics, Decision-Enhancement, and Analytic Sciences) Center, Veterans Affairs Salt Lake City Health Care System, Salt Lake City, UT USA; 7 Division of Epidemiology, School of Medicine, University of Utah, Salt Lake City, UT USA; 8 Department of Medicine, David Geffen School of Medicine, University of California, Los Angeles, Los Angeles, CA USA; 9 John R. and Teresa Lozano Long School of Medicine, UT Health San Antonio, San Antonio, TX USA

**Keywords:** Substance use disorders, Permanent supportive housing, Homelessness, Implementation science, Veterans

## Abstract

**Background:**

Homeless-experienced Veterans (HEVs) have higher rates of substance use disorders (SUDs) than housed Veterans, which impairs their ability to retain housing. The Department of Housing and Urban Development-VA Supportive Housing (HUD-VASH) initiative, which provides subsidized permanent housing and supportive services, contributed to the 50% reduction in Veteran homelessness over the past decade. However, ~ 40% of Veterans exit HUD-VASH within two years, often due to untreated SUDs. We will use two strategies to support the implementation of Medications for Addiction Treatment (MAT) and Cognitive Behavioral Therapy for Substance Use Disorders (CBT-SUD) in 12 HUD-VASH sites; conduct an evaluation of this implementation effort; and generate an implementation playbook to support continued spread of MAT and CBT-SUD in HUD-VASH.

**Methods:**

We will use Replicating Effective Programs (REP) to implement MAT and CBT-SUD at 12 sites over 18 months. After 9 months of REP alone, half (n = 6) of these sites will also receive Consumer Engagement (CE) for 9 months, activating HEVs to adopt these practices via peer coaching. We will conduct a type 3 hybrid cluster-randomized trial to compare the impacts of REP versus REP + CE. Randomization will occur at two levels: implementation start date (3 cohorts) and the implementation strategy (REP versus REP + CE). We will use stratified block randomization to balance site size among sites receiving each strategy across cohorts. We will use mixed methods to assess the impacts of REP versus REP + CE on implementation outcomes (reach [primary outcome], adoption, and sustainment); Veteran outcomes (primarily housing); provider and Veteran experiences; and costs and budget impacts. We hypothesize that REP + CE will have higher implementation costs than REP but result in improved MAT and CBT-SUD implementation and Veteran outcomes, leading to a business case for REP + CE.

**Discussion:**

Implementing MAT and CBT-SUD within HUD-VASH can improve HEVs’ housing and health. By identifying effective strategies to support the implementation of these practices, we aim to inform other implementation efforts of behavioral health practices in homeless service settings.

**Trial registration:**

This project was registered with ClincialTrials.gov as “Coordinated Access for Addiction Recovery and Equity in VA Supportive Housing.” Trial registration NCT07141394, registered 8/26/2025 (https://clinicaltrials.gov/study/NCT07141394?term=CARE-VASH&rank=1).

Contributions to the literature

- This protocol uses tailored and data-driven implementation approaches to address inequities in the use of evidence-based practices for substance use disorders.
- This trial integrates a cluster-randomized design and mixed-methods evaluation to compare the impacts of a base versus enhanced implementation strategy on the implementation and effectiveness of two evidence-based substance use disorder practices.
- This study uses a robust methodologic framework to address disparities in the implementation of substance use disorder interventions in a complex, highly field-based social service setting.

## Background

Homeless-experienced Veterans (HEVs) have higher rates of substance use disorders (SUDs) than their housed counterparts [1], which impair their ability to retain housing. The Department of Housing and Urban Development-VA Supportive Housing (HUD-VASH) initiative—which provides subsidized, permanent housing and supportive services—has contributed substantively to the 50% reduction in Veteran homelessness over the past decade [2]. The program has housed ~180,000 Veterans since 2012 [3]. Yet, up to 40% of Veterans exit HUD-VASH within two years of housing [4]. Most of these exits represent returns to homelessness, with untreated SUDs as a common primary or contributing factor [5–8]. National implementation of evidence-based practices (EBPs) for SUDs within HUD-VASH would substantively advance VA’s commitment to end Veteran homelessness.

HUD-VASH is a “Housing First” program, in which Veterans receive independent, permanent housing with supportive services [9]. Housing First increases days in stable housing and decreases Emergency Department (ED) use and hospitalizations, without worsening SUDs [9]. Each HUD-VASH team is assigned to a VA medical center or clinic (i.e., VA healthcare system division); teams are interdisciplinary, led by a social worker and staffed by case managers, nurses, and peer supports, and serve Veterans largely in the field (e.g., home visits).

In our prior evaluation [10] for HEVs who attained HUD-VASH housing from 2018–2021 (n = 63,939), most had ≥ 1 SUD (66%, n = 42,182). Veterans with SUDs were 1.4 times more likely to have a negative exit from HUD-VASH within one year of move-in. Though nearly half (47.3%, n = 19,931) of HEVs with SUDs in HUD-VASH had ≥ 1 SUD specialty visit, receipt of EBPs for SUDs was low. In the year after HUD-VASH move-in, only 9.6% and 19.9% of Veterans with alcohol use disorder (AUD) and opioid use disorder (OUD), respectively, received ≥ 1 medication for addiction treatment (MAT) prescription [11]. This finding is concordant with a prior study of OUD in VA homeless programs, which describes gaps in MAT provision and asserts the importance of policy goals for addressing OUD among HEVs [12]. Moreover, among HEVs with stimulant use disorder (35.9% of HUD-VASH participants), < 0.5% received Cognitive Behavioral Therapy for SUDs (CBT-SUD) [10]. Therein, though the VA has implemented MAT and CBT-SUD within mental health clinics, HUD-VASH adoption is low. National implementation of evidence-based practices (EBPs) for SUDs within HUD-VASH can improve housing outcomes among homeless-experienced Veterans.

This paper describes the Coordinated Access for Addiction Recovery and Equity in VA Supportive Housing (CARE-VASH) Quality Enhancement Research Initiative (QUERI), which will implement MAT and CBT-SUD at 12 HUD-VASH sites and compare the impacts of two implementation strategies on implementation and effectiveness outcomes. To implement and sustain MAT and CBT-SUD across sites, we will use the Replicating Effective Programs (REP) bundle for 18 months, engaging diverse stakeholders (“Learning Community”) to package the EBPs; provide staff training; conduct evaluation; and support maintenance [13]. In addition to REP, 6 sites will receive 9 months of Consumer Engagement (CE). In CE, Veterans will be activated to use EBPs via a Veteran panel, educational materials, and HUD-VASH peer coaching.

Partnered with VA mental health and homeless service leaders, our Specific Aims are to: 1) Use REP and REP + CE to support the implementation and sustainment of MAT and CBT-SUD in 12 HUD-VASH sites; 2) Compare, in a type 3 hybrid implementation-effectiveness trial, the impacts of REP versus REP + CE on MAT and CBT-SUD reach, adoption, and sustainment; Veteran outcomes and provider experiences; and costs and budget impacts; and 3) Generate an implementation playbook for program partners to support continued spread and sustainment of MAT and CBT-SUD in HUD-VASH.

## Methods

### Overview

We will conduct a cluster-randomized trial comparing sites (or clusters) randomized to REP versus REP + CE. Specifically, we will conduct a type 3 hybrid implementation-effectiveness trial [14] using a stepped wedge design with randomization at two levels: implementation start date (3 cohorts) and implementation strategy (REP vs. REP + CE). We will use stratified block randomization to balance site size among sites receiving each strategy across cohorts. We will use mixed methods to assess the impacts of REP vs. REP + CE. We hypothesize that REP + CE will have higher implementation costs than REP, but result in improved and more equitable EBP reach, adoption, sustainment, and housing outcomes, leading to a business case for REP + CE.

### Conceptual framework

**Fig. 1:**
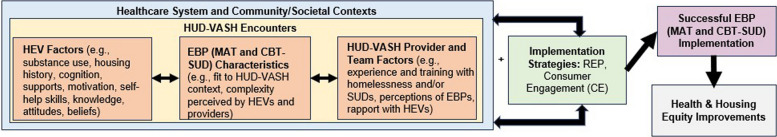
Health equity implementation framework (HEIF), specified for MAT and CBT-SUD implementation in HUD-VASH. HEV = Homeless-experience veterans. EBPs = Evidence-based practices. REP = Replicating effective programs. MAT = Medications for addiction treatment. CBT-SUD = Cognitive behavioral therapy for substance use disorders

CARE-VASH is guided by the Health Equity Implementation Framework [15] (HEIF, Fig. 1), which identifies how contextual and societal factors influence EBP implementation and address disparities. HUD-VASH encounters are conceptualized as the confluence of interactions between HEVs, EBPs, and HUD-VASH teams. These interactions are nested within healthcare system and community-level contexts. These factors interplay with strategies that support EBP implementation to enhance equity by addressing disparities created by structural inequities.

### Medications for Addiction Treatment (MAT)

Medications for Alcohol Use Disorder (MAUD) and Medications for Opioid Use Disorder (MOUD) are implementation priorities within VA primary care and mental health but have not been systematically incorporated in HUD-VASH. MAUD includes naltrexone and acamprosate. Across meta-analyses and systematic reviews, MAUD resulted in significantly decreased alcohol consumption compared to placebo [16, 17]. In a meta-analysis [16] of 118 randomized controlled trials (RCT), the number needed to treat (NNT) to prevent return to drinking was 11 for acamprosate and 18 for oral naltrexone. Injectable naltrexone was significantly associated with reduced drinking days (weighted mean difference [WMD] −5 days).

MOUD includes methadone, buprenorphine, and extended-release naltrexone. In a meta-analysis of 11 RCTs [18], methadone was significantly more effective than placebo or non-pharmacological treatments on treatment retention (risk ratio [RR] 4.44) and the reduction of morphine-positive urine or hair analysis (RR 0.66). In a meta-analysis of 31 RCTs, buprenorphine was significantly more effective than placebo on treatment retention at low (RR 1.5); medium (RR 1.74); and high (RR 1.82) doses. In an RCT [19] comparing extended-release naltrexone to counseling, relapse was significantly lower in the naltrexone group (43% vs. 64%) over 24 weeks. Buprenorphine and methadone significantly reduce mortality [20], opioid-related overdoses and acute care [21].

### Cognitive Behavioral Therapy for Substance Use Disorders (CBT-SUDs)

CBT-SUD is an evidence-based psychotherapy used across SUDs [22, 23]. It is delivered in 12–16 sessions by mental health providers in individual or group sessions (in-person or virtual), addressing maladaptive interactions among thoughts, emotions, and substances [24]. A meta-analysis and systematic review demonstrate that CBT-SUD reduces frequency and quantity of use [25, 26]. Among Veterans, CBT-SUD is associated with improvements in personal relationships [27].

The strength of evidence for CBT-SUD is bolstered by evidence demonstrating its mechanism of action (e.g., improved self-efficacy). In VA’s CBT-SUD Training Program (in specialty mental health, not homeless programs), 457 Veterans enrolled in CBT-SUD [24]. Among these Veterans, intent-to-treat analyses showed > 50% reduction in heavy drinking and other drug use; 29% reduction in craving days; 47% reduction in substance use related problems; and improvements in quality of life.

### Implementation strategy: Replicating Effective Programs (REP)

CARE-VASH QUERI will use REP at 12 HUD-VASH sites to implement MAT and CBT-SUD. REP engages stakeholders to package EBPs and their associated training materials; it is the go-to implementation strategy for VA mental health EBPs but has not been used routinely in HUD-VASH [24]. Our use of REP is intended to enhance HUD-VASH providers’ skills and clinical competency, enhancing MAT and CBT-SUD reach, adoption, and sustainment. REP is well-suited for EBP implementation in HUD-VASH; adapting and packaging EBPs and associated trainings for program and population contexts, using stakeholder input, is critical for effective implementation in homeless service settings [28].

Table 1 summarizes REP’s phases in CARE-VASH. In REP phase one (“pre-conditions”), completed prior to this proposal, MAT and CBT-SUD were identified as preferred EBPs for implementation in meetings with our national VA mental health and homeless program partners. The first year of CARE-VASH will encompass REP phase two (“pre-implementation”) and will engage a Learning Community (national, interdisciplinary, and multi-level stakeholders, including HEVs with SUDs, providers and managers, and regional/national representatives from our partners). We will work with the Learning Community to package training materials; orienting HUD-VASH sites to these EBPs and planning for logistics; and assessing and refining process and outcome metrics for our evaluation. Nationally, our VA mental health partner has training paradigms for both EBPs: academic detailing (AD) for MAT and technical assistance (TA) for CBT-SUD. As these are considered the lowest level of implementation support that can result in adequate MAT and CBT-SUD adoption, we will use REP to adapt AD and TA to fit the needs of HUD-VASH. REP phase three (“implementation”) includes delivery of the MAT and CBT-SUD packages to 12 HUD-VASH sites, with refinement informed by our evaluation [29]. REP phase four (“maintenance”) will be iterative throughout CARE-VASH, anchored in our evaluation, including assessing financial factors and organizational changes needed for EBP sustainment. We will engage the Learning Community to develop a final implementation playbook, including strategies for ongoing evaluation and improvement, that can be used for national spread and sustainment by our partners.

**Table 1:** Replicating effective program (REP) phases, specified for CARE-VASH

Pre-conditions	Pre-implementation	Implementation	Maintenance
Select EBPs, identify implementation barriers, and engage Learning Community to draft implementation packages (e.g., adapting Academic Detailing [AD] for MAT; CBT-SUD training & technical assistance [TA])	Develop implementation packages, using AD and TA, with the Learning Community; orient sites to EBP implementation; refine process and outcome metrics	Train and fill knowledge gaps among HUD-VASH staff; conduct implementation evaluation; refine both EBPs’ implementation packages	Re-organize packages for both EBPs with Learning Community, aiming for EBP spread and sustainment, identifying organizational and financial prerequisites for spread

#### REP specified for MAT implementation

Since low MAT adoption has been attributed in part to clinicians’ knowledge and training gaps—and AD trains clinicians in the use of novel evidence-based medications—AD will be incorporated into REP for MAT. AD uses 1:1 or small group training, audit-and-feedback tools, and clinician-targeted handouts to influence clinicians to use EBPs. These materials will be available on an online CARE-VASH toolkit once finalized. Current VA dashboards (detailing eligible patients by provider and by division) for MAT [30] will be adapted for HUD-VASH during REP pre-implementation. These dashboards will support educational outreach to prescribers (physicians and physician extenders) who treat HEVs in HUD-VASH with AUD and OUD. Outreach will be delivered by pharmacists trained in AD and with expertise in SUDs. Though the frequency and content of outreach will be determined during pre-implementation, we anticipate individual and small group virtual sessions with prescribers, employing scripts and messaging tailored for CARE-VASH. To enable sustainment, prescribers will be able to receive training from these pharmacists throughout CARE-VASH maintenance. Prescribers will use MAT with HEVs as part of their routine care of HEVs, which encompasses in-person and virtual individual visits, in the clinic or in the field (e.g., home visits). MAT is ideally delivered longitudinally as AUD and OUD are chronic illnesses.

#### REP specified for CBT-SUD implementation

Connecting intensive formal training to longer-term TA can enhance psychotherapy implementation [31, 32]. The specifics of CBT-SUD training and TA processes will be refined during pre-implementation; we anticipate that HUD-VASH case managers (social workers) who desire CBT-SUD training will first receive intensive CBT-SUD training: an asynchronous, individual web-based training from VA’s CBT-SUD mental health roll-out; and a one-day virtual, small group intensive CBT-SUD workshop on VA’s CBT-SUD protocol [31]. We anticipate that materials used for training will include existing VA videos and electronic materials available on internal VA servers along with adapted training materials (e.g., manuals, presentations) housed on VA servers through CARE-VASH. TA will follow, with weekly 1-h virtual small group case consultation sessions (for 4 months) moderated by CARE-VASH consultants who are social workers experienced in CBT-SUD to a train-the-trainer level. Case managers will deliver weekly CBT-SUD in individual or group sessions to HEVs on their caseloads for ~12 weeks, in person or virtually. Consultants will train case managers to attain fidelity via the CBT-SUD Rating Scale [31], defined as ≥ 80% fidelity for ≥ 6 consecutive sessions with ≥ 2 HEVs. Consultants will be available for as-needed virtual individual case consultation throughout REP. They will moderate virtual small group “booster consultations” (at 12 and 18 months) that use self-reflection to address drift away from high-fidelity practice [33]. During REP’s maintenance phase, all trained case managers and the consultants will join a Microsoft Teams group for asynchronous case discussion.

###  Implementation strategy: REP + Consumer Engagement (CE)

In addition to REP, 6 of the 12 sites will receive 9 months of CE, activating HEVs to enhance EBP adoption and sustainment. CE has been successfully used within VA in a mental health implementation effort [34]; it can increase trust among Veterans to mitigate disparities [14] for socially vulnerable groups. Particularly for these EBPs—which have well-established national VA behavioral health rollouts, but low HUD-VASH penetrance—we hypothesize substantive value in engaging HEVs to become active participants in HUD-VASH encounters. All CE will be led by trained HUD-VASH peer support specialists.

Table 2 depicts key components of CE [34]. We will develop a panel of Veterans in HUD-VASH in recovery from SUDs (n ~ 8–10), embedded within our Learning Community. We will recruit HEVs via HUD-VASH peer support leaders at participating sites. We will use gift cards to compensate panel members for all activities. During pre-implementation, we will work with the panel to refine existing Veteran-facing MAT and CBT-SUD materials, ensuring appropriateness to HEVs’ experiences and backgrounds. These materials will be available on the online CARE-VASH toolkit. We will work with the panel to develop materials to train HUD-VASH peers—building on their lived SUD expertise—to activate HUD-VASH Veterans to seek MAT and CBT-SUD from their providers at REP + CE sites. Over the implementation period, peers will work with HEVs with SUDs who are disengaged from SUD care, providing coaching and education. We anticipate engaging peers in two interactive virtual training sessions (2 h each) on CE. After CE launches, we plan biweekly virtual learning collaboratives (60 min/group session) over 9 months, to foster knowledge sharing among peers regarding coaching and CE. To plan for sustainment, we will finalize our CE package, including HUD-VASH peer and Veteran toolkits.

**Table 2:** Consumer engagement (CE), specified for CARE-VASH

Involve HEVs in implementation activities	Intervene with HEVs to enhance adoption and adherence to EBPs	Prepare HEVs to be active participants in HUD-VASH	Increase HEV demand for EBPs	Disseminate information about innovations
Build HEV panel, embedded in Learning Community, to inform CE strategy	Work with HEV panel and Learning Community to refine HEV-facing educational materials about EBPs; train peers	Peers activate HEVs to engage in HUD-VASH encounters around SUD services	Peers coach HEVs about MAT and CBT-SUD; distribute HEV-facing materials	Finalize CE package, including peer and Veteran toolkit, with Learning Community input (including HEVs)

### Participating sites

We identified 12 geographically diverse HUD-VASH implementation sites; each is nested in a division of a VA healthcare system and is comprised of ≥ 1 HUD-VASH team. Collectively, these sites serve ~7908 HEVs/year. Each site will implement MAT and CBT-SUD. Of note, our sample size (12 sites) was pre-determined by our funder.

### Study design

We plan a type 3 hybrid implementation-effectiveness trial [14], registered as NCT07141394 on 8/26/2025 and determined to be non-research by VA’s Central Institutional Review Board. We will use stratified block randomization, stratified by tertiles of each site’s number of HUD-VASH full-time equivalent (FTE) employees (proxy for size and resources).

**Fig. 2:**
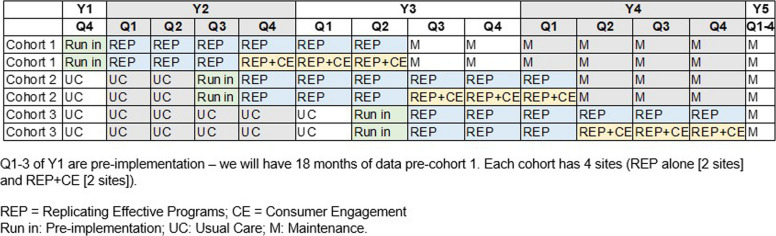
Stepped wedge cluster randomized trial to assess implementation outcomes

**Fig. 3:**
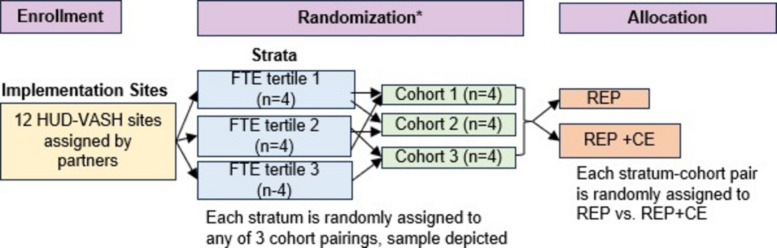
Modified CONSORT diagram describing stratified block randomization. *We will use stratified randomized, stratified by tertiles of each site’s number of HUD-VASH full-time equivalent (FTE) employees (proxy for size and resources). REP = Replicating Effective Programs; CE = Consumer Engagement

Within the 12 sites, we will use a stepped wedge cluster randomized trial design [35] (Fig. 2) to study implementation outcomes, balanced for allocation to treatment timing and assignment to REP vs. REP + CE arms across 3 strata of 4 sites each (Fig. 3). Using a random number generator with assignment by our statistician, each stratum will be randomly assigned to 1 of 3 possible cohort pairings (cohorts 1 & 2; 2 & 3; 1 & 3). Within each stratum, 4 sites will be randomly assigned to 1 of the 2 cohorts in the pair, ensuring that there are early and late treatment timings within each stratum. Within each stratum-cohort combination (n = 2), the REP vs. REP + CE implementation strategy arm will be randomly assigned, and consent obtained thereafter. The staggering of sites into cohorts is necessary to provide HUD-VASH sites with optimal EBP and CE training within our resources. We selected the 9-month duration of REP, before augmenting with 9 months of CE at REP + CE sites, because we found this duration of REP to be effective in prior trials of a psychosocial EBP for HEVs [36]. We will be unable to blind sites (clusters) assigned to CE augmentation, as we need to engage with leadership regarding peer supports’ commitments. To optimize power in our effectiveness analyses, we will compare sites that receive REP vs. REP + CE vs. all other HUD-VASH sites in the nation that are not engaged in CARE-VASH.

### Evaluation

We will evaluate our EBPs’ implementation and effectiveness, with the goals of: 1) capturing fidelity to REP and CE; 2) comparing MAT and CBT-SUD reach, adoption and sustainment achieved by REP and REP + CE, and between REP vs. REP + CE; 3) comparing Veteran outcomes (housing stability, acute care use) and Veteran and provider experiences achieved by the strategies and EBPs; and 4) comparing costs and budget impact for EBPs and strategies. Below, we describe the methods for each goal, followed by methods for qualitative data collection and analyses that cut across goals. Of note, if we receive a request for access to our evaluation data, we will establish a Data Use Agreement (DUA). The DUA will enable a limited dataset, excluding all identifiers, and prohibiting the recipient from identifying or re-identifying any individual whose data are included.

#### Fidelity to REP and CE

Fidelity to REP and CE will be assessed in three domains [37]: adherence (extent to which strategies took place); dose (amount of each strategy received and proportion of providers and HEVs who received each strategy); and responsiveness (receptivity and involvement of providers and HEVs to strategies).

We will maintain an Implementation Activity Log that summarizes all project activities pertaining to REP and CE. The log will include dates and times of all training and TA (across both EBPs); attendees; and open-ended, templated notes on the implementation process. All CE encounters by HUD-VASH peers will be recorded in a templated Electronic Health Record (EHR) note with checkboxes for activities performed [38] and space for free text about CE content. We will abstract data from these notes to augment the Implementation Activity Log. Adherence to implementation strategies will be assessed by comparing this log and abstracted data to processes described in the implementation strategies above. To assess adherence to REP specific to MAT, we will use an existing “advanced skill training fidelity tool” to assess adherence to core components of AD. For CBT-SUD, consultants will record TA activities in the Implementation Activity Log.

Dose will be captured by describing the frequency of training/TA and CE delivered to each site (and % of eligible participants attending). Responsiveness will be characterized by analyzing data from open-ended log notes (including free text in peer notes) and periodic reflections (brief, ethnographically informed, guided discussions) [39] with HUD-VASH supervisors that capture implementation context; and interviews with HUD-VASH staff at 18 months post-implementation.

#### MAT and CBT-SUD reach, adoption, and sustainment

Table 3 lists measures for reach, adoption, and sustainment. Measures of reach for MAT indicate one prescription (30–90 days/duration) and use of MAT for 180 days [40]. For CBT-SUD, reach assesses a single visit and a threshold of 9 visits (which may equate to meaningful impacts in substance use/functioning) [41]. We will use VA administrative data to abstract these measures for HEVs in HUD-VASH at implementation sites. Sustainment will reflect these reach and adoption measures after our intensive implementation supports end (cross-sectionally at 9 months after we end each implementation cohort).

**Table 3:** Reach, adoption, and sustainment measures

	**MAT**	**CBT-SUD**
Reach^*^	• % HEVs who used MOUD for OUD, ≥ 1 prescription • % HEVs who used MOUD for OUD for 180 days since initial prescription (without a break ≥ 14 days in supply) • % HEVs who used MAUD for AUD, ≥ 1 prescription • % HEVs who used MAUD for AUD for 180 days since initial prescription (without a break ≥ 14 days in supply)	• % HEVs with SUD with ≥ 1 CBT-SUD visit • % HEVs with SUD with ≥ 9 CBT-SUD visits
Adoption^*^	• % HUD-VASH and mental health prescribers engaged with HEVs^**^ who ordered MOUD/MAUD at least once in the Electronic Health Record	• % HUD-VASH case managers who delivered ≥ 1 CBT-SUD session
Sustainment^¶^	• Reach and adoption measures described above	Reach and adoption measures described above

^*^Reach and adoption measures will be assessed at 18 months post-implementation

^**^Mental health prescribers engaged with HEVs will be defined as prescribers listed on AD dashboards, i.e., most recent mental health prescriber for Veterans who had ≥ 2 billable HUD-VASH encounters in the past year (§B3bi)

^¶^Sustainment will be assessed 9 months after we conclude REP vs. REP + CE for each cohort. HEVs referenced in this table include HEVs enrolled in HUD-VASH at implementation sites

We will use a staggered adoption model [42], comparing reach and adoption pre-CARE-VASH, to REP alone, and to REP + CE. These models utilize fixed effects for sites to account for time-invariant confounders between sites as well as fixed effects for time-period to account for secular trends. We will implement this approach [42] using a doubly robust estimator based on stabilized inverse probability weighting [43], with weights based on HEV demographics and comorbidities (using the Multimorbidity-Weighted Index) [44]. The average treatment effect on the treated will be estimated based on comparison to the not-yet-treated periods and presented using an event-study approach which will estimate the intervention effect across time (i.e., for each quarter-year). In addition to providing intervention estimates for each quarter-year of the study, a pooled intervention effect estimate will be aggregated across the study period by taking weighted averages of the event-study estimates with non-parametric bootstrapping (1000 replications) used to provide standard errors for aggregated estimates. Sustainment will be compared between sites that received REP vs. REP + CE, with reach and adoption measures adjusted as described.

#### Sample size and power

Across the 12 sites, we will have 7908 HEVs undergoing REP or REP + CE. For assessing reach, we will examine the proportion of eligible HEVs who receive MAT and the proportion of eligible HEVs who receive CBT-SUD. Among HUD-VASH Veterans with AUD (53%) and OUD (16%), ~ 9.9% and 19.9% currently receive MAT, respectively. Over half (66%) are eligible for CBT-SUD, though only 0.5% receive it. We can approximate the power of our staggered adoption model approach to compare REP alone (or REP + CE) to pre-CARE-VASH based on the power to detect a difference in proportion assuming a within-site correlation of outcomes over time of rho = 0.04. With this sample size using a two-sided alpha level of 0.05, we would have 80% power to minimally detect an absolute increase in reach for MAUD of 2.7 percentage points, MOUD of 6.6 percentage points, and CBT-SUD of 0.8 percentage points. These minimally detectable estimates are much smaller than those found for a MAUD implementation effort [45] and an MOUD implementation study [46]. Though a systematic review of CBT implementation studies note that the implementation evaluations did not address changes in reach [47], we can extrapolate from MAT studies to estimate that concerted implementation efforts would achieve an increase in CBT-SUD’s reach of > 0.8 percentage points.

### Veteran outcomes and provider experiences

#### Housing stability

We will use VA administrative data to assess housing outcomes for HEVs at our implementation sites. We conceptualize housing stability as our primary effectiveness outcome, strongly associated with substance use frequency and intensity [5–8], which are salient expected outcomes of both EBPs that cannot be reliably abstracted from VA administrative data. We will partner with the Scientific Approaches to Improving Housing and Economic Security Outcomes Advanced by a Learning Health System through Data driven Evaluation and Research (SHELTER) [48], which used natural language processing (NLP) and structured EHR data to build a common longitudinal dataset of HEVs’ housing situations, clinical characteristics, and other social determinants of health. This dataset combines elements across VA and non-VA data pertaining to homelessness and provides longitudinal housing data for all HUD-VASH Veterans available for research studies.

Impacts on housing will be defined as a difference in the mean days homeless over 1 year of follow-up for HEVs enrolled in CARE-VASH sites that received REP vs. REP + CE, versus the expected number of days homeless had they enrolled in a comparison group of all sites not engaged in CARE-VASH. A binary indicator of homelessness will be extracted from SHELTER each day there is EHR documentation of housing status. We will fit a logistic regression model, with an indicator of site assignment (comparison group, REP, REP + CE) as an independent variable, while controlling for HEV characteristics, co-morbidities, temporal trends in the risk of homelessness, and variation across sites. To account for irregular EHR documentation of housing status and possible imbalance between HEVs retained in HUD-VASH vs. those who exited and became homeless, the model will be weighted using inverse intensity weighting (IIW); IIW reduces bias induced by outcome-dependent assessment times in longitudinal studies [49–51] and has precedent in studies leveraging EHR data to evaluate VA homeless programs. For each HEV, the weighted model will be used to estimate the counterfactual probability of being homeless each day over 1 year follow-up for the REP, REP + CE, and the comparison group of sites not engaged in CARE-VASH [52–54]. The expected number of days homeless for each group will be calculated as the sum of the daily probabilities. The treatment effects for each level of intervention will be estimated as the mean difference in the expected number of days homeless had the HEV enrolled in a site with REP or REP + CE, respectively, compared to the expected number of days homeless had they enrolled in a comparison group site. Non-parametric bootstrapping will be used to calculate simultaneous 95% confidence intervals for the two treatment effects.

#### Acute care use

We anticipate that HEVs who receive MAT and/or CBT-SUD will have decreased ED visits and hospitalizations associated with primary SUD diagnoses. We will use the VA Managerial Cost Accounting (MCA) database to compare use of these services among HEVs in HUD-VASH at sites receiving REP vs. REP + CE; and among HEVs at CARE-VASH vs. comparison sites. Differences in acute care use among HEVs at REP, REP + CE and comparison sites will be examined using negative binomial regression models with offsets for the time period each HEV is under observation.

#### Veteran and HUD-VASH staff experiences

We will conduct semi-structured interviews with HEVs with SUDs at implementation sites at baseline and 18 months, characterizing and comparing HEVs’ experiences of and satisfaction with HUD-VASH and SUD services, and factors influencing those experiences, pre- and post-EBP implementation; 18-month interviews will also assess HEVs’ experiences with CE and perceived impacts at sites with the REP + CE strategy. We will use semi-structured interviews with HUD-VASH staff at implementation sites at baseline and 18 months to characterize provider experiences with REP and peers’ experiences with CE. Interviews will also assess experiences of and satisfaction with providing HUD-VASH services to HEVs with SUDs pre- and post- implementation, as well as factors that influence those experiences. We will assess differences in staff experience between sites that received REP vs. REP + CE, stratifying experiences by valence. Interviews will be integrated with periodic reflections to assess contextual and strategy factors associated with HEV and staff experiences.

#### Economic evaluation

We will assess the costs, cost offsets, and non-financial benefits of the EBPs’ sustainment and spread in HUD-VASH; and the budget impact of REP vs. REP + CE.

##### Assessing MAT and CBT-SUD Costs as actually implemented

The predominant cost of delivering MAT and CBT-SUD in HUD-VASH programs is prescriber time and case manager time, respectively. We will use VA administrative data to identify visits in which MAT was prescribed or CBT-SUD was delivered. Interviews with HUD-VASH prescribers will be used to estimate minutes/visit spent on MAT; at implementation sites, we will add a mandated field to templated CBT-SUD notes that indicates time spent outside the encounter. We will multiply employee hourly wages by average time spent on each EBP. Data from 18-month HUD-VASH staff interviews will be used to glean contextual factors that may influence clinical and case manager activities.

##### Costs of implementation strategies

We will use the Implementation Activity Log to draft a list of tasks performed by each CARE-VASH implementation team member. Prior to cohort 3, implementation team members will refine and finalize their task lists; we will input these tasks into software which allows each team member to submit weekly time logs customized to match their task list, recording daily time spent on each task, with the ability to also specify tasks not on the pre-specified list. We will link these data to team members’ hourly costs. To decrease time tracking burden among academic detailers who are engaged in the strategy but external to CARE-VASH, we will use a brief cost capture template at 9 months post-implementation. This template delineates time spent on specific clinical activities and is derived from a practical guide to costing behavioral interventions [55]; the web-based form that captures the template will also query these detailers’ location and hourly salary/fringe. Peers engaged in CE will report time spent/week in a Microsoft Teams poll administered during learning collaboratives.

##### Downstream costs

Both EBPs may lead to changes in VA healthcare use. We will consider all non-intervention-related healthcare use as downstream (VA outpatient, pharmacy, and inpatient care). Costs of this downstream utilization will be captured using the VA MCA.

##### Budget impact analysis

We will also prepare a budget impact analysis (Table 4), characterizing the costs, cost offsets, and non-financial benefits of: MAT and CBT-SUD sustainment and spread in HUD-VASH; and REP vs. REP + CE. We will compare healthcare utilization costs between REP and REP + CE sites using a difference-in-differences research design. This will be performed using both the standard two-way fixed effect approach as well as with the Callaway and Sant’Anna [42] estimator, which takes into account staggered adoption. Our cost analyses will provide data about the magnitude and features of expected EBP costs and benefits. We will assess whether REP + CE yields a sufficient combination of downstream financial cost offsets and non-financial benefits to justify REP + CE vs. REP alone in spreading and sustaining MAT and CBT-SUD. We will also provide information about HUD-VASH site-level contextual factors that may signal an above-average benefit from using REP + CE (vs. REP alone).

**Table 4:** Budget impact analysis

Expected resources (costs)		Expected benefits
**Implementation Strategy Costs**	**Care delivery costs**	**Cost offsets and non-financial benefits**
• *Fixed*: Implementation team, HUD-VASH and mental health staff time spent in EBP training • *Variable*: Staff time spent giving/receiving AD and/or CBT-SUD TA, implementation team time spent consulting with HUD-VASH peers, peer time spent delivering CE*	• HUD-VASH and mental health prescriber time spent delivering MAT • HUD-VASH staff time spent delivering CBT-SUD • Downstream healthcare use	• *Cost offset:* decreased use of VA housing services • *Non-financial benefits*: HEVs’ housing stability; improved HEVs’ care experiences; improved HUD-VASH team experiences providing care

### Qualitative data collection

#### HUD-VASH staff semi-structured interviews

We will interview (45 min/interview) HUD-VASH prescribers (~12, 4/cohort, 1/implementation site), case managers (~36, 12/cohort, 3/implementation site) and peers (~18, 6/cohort, 3/REP + CE site) engaged in EBP implementation. Interviews will be conducted, by cohort, at baseline and at 18 months post-implementation. Interview guides will be grounded in the HEIF to assess at baseline: staff background; SUD practices; contextual factors; and experiences of EBP training. Interviews at 18 months will assess experiences with the EBPs and strategies; time spent delivering EBPs; recommendations for EBP implementation support; and barriers of and facilitators to EBP reach, adoption, and sustainment.

#### Veteran semi-structured interviews

HEVs with SUDs at participating HUD-VASH sites will take part in interviews (30–45 min/interview) at baseline and 18 months post-implementation. We will use VA’s homeless registry to identify ~36 HEVs with SUDs (12/cohort) who were enrolled in HUD-VASH in the year prior to implementation, ensuring that about half have AUD and/or OUD (i.e., MAT-eligible). We will interview 36 distinct Veterans (12/cohort) enrolled in HUD-VASH between 12–18 months after implementation, all of whom received one or both EBPs. We will use maximum variation sampling [56] to ensure diversity by site and SUD diagnosis; we will oversample women to ensure their representation. Interviews will assess perceived needs and experiences of SUD services within HUD-VASH; for HEVs at sites that received REP + CE, we will query about experiences with and perceptions of CE.

#### Periodic reflections

Periodic reflections will be conducted monthly (15–30 min/each) with MAT and CBT-SUD Implementation Teams (n = 6), and quarterly with a HUD-VASH supervisor who works most closely with staff trained in our EBPs and peers trained in CE (n = 12, 4 per cohort).

#### Analyses of qualitative data

All interviews will be recorded and transcribed; we will take detailed notes on reflections. All qualitative data will be analyzed using rapid analysis methods [57, 58]. We will create structured summaries for each interview or reflection using templates organized by domains of interest within our conceptual framework; we will develop matrix displays to synthesize content by site, cohort, and participant, before completing summary tabulation tables to validate data patterns. Targeted in-depth coding and analysis using ATLAS.ti will be conducted as needed, using the constant comparison method [59] to confirm/disconfirm exploratory hypotheses and/or explore emergent data. Qualitative and quantitative data will be gathered concurrently over the course of implementation [60, 61] and integrated to identify how contextual factors in qualitative data are associated with quantitative outcomes.

### Products to support continued spread and sustainment of MAT and CBT-SUD nationally

Though the scope of CARE-VASH is to implement and evaluate MAT and CBT-SUD in 12 HUD-VASH sites, our goal is for our partners to use knowledge gained to implement and sustain these EBPs nationally. We will package the findings and products from this program into an implementation playbook [62, 63], a user-friendly compendium of implementation processes, targets, and outcomes for MAT and CBT-SUD spread and sustainment. The playbook will be developed iteratively and collaboratively over the implementation period, in dialogue with our sites, partners, and Learning Community, to ensure optimal match between content, program office, and site needs. It will be sufficiently detailed to serve as a how to guide for spread and sustainment. The centerpiece for each playbook will be the finalized EBP packages with products that have been, or will be, generated across all phases of REP and REP + CE.

## Discussion

Enhancing the implementation of evidence-based SUD services for HEVs will address one of this group’s most salient predictors of returns to homelessness. Widespread implementation of MAT and CBT-SUD in HUD-VASH hold potential to improve HEVs' housing and health. As notable inequities by sociodemographic factors pervade access to and use of EBPs for SUDs [64, 65] tailored, data-driven implementation is valuable to address these disparities. This project aims to identify effective practices to support the implementation of EBPs for SUDs in a complex, field-based context, and to reduce disparities faced by HEVs.

We will use REP to address provider factors that impact MAT and CBT-SUD implementation across 12 sites. In some settings and contexts, REP alone may be adequate to enable MAT and CBT-SUD implementation. However, REP alone does not fill knowledge and motivation gaps that may impede HEVs from engaging in effective SUD services, which can be addressed with CE. By starting each of our implementation cohorts with REP alone (for 9 months), then augmenting (at REP + CE sites) with 9 months of CE, we enable within-site analyses for implementation outcomes [42]; we also contribute to the science surrounding the selection of best strategies to support the adoption of complex practices in homeless service settings. Though we anticipate barriers to implementation (e.g., staff turnover, staff training gaps) in this project, our dynamic base implementation strategy, grounded in stakeholder engagement and context-specific tailoring, is well-suited for homeless programs [28].

We note that this implementation and evaluation project was developed within VA, with close collaboration from mental health and HUD-VASH partners, and may not extrapolate to non-VA settings or other VA homeless programs. Rather, we aim to produce products that allow for continued spread of MAT and CBT-SUD within HUD-VASH. We also recognize that our selected implementation strategies are costly and time-intensive, and may prove challenging in community-based, low-resourced settings.

Despite these limitations, for HUD-VASH Veterans, increased adoption of EBPs for SUDs has potential to improve housing, health, and quality of life. MAT and CBT-SUD have precedent for concerted implementation efforts in VA mental health; these practices can benefit HEVs in sustaining exits from homelessness. The products planned for our partners will help to facilitate spread beyond this study. Increasingly our understanding of effective practices to support MAT and CBT-SUD implementation in complex social service settings can support the implementation of other evidence-based behavioral health practices in homeless programs within and outside VA.

## Data Availability

No datasets were generated or analysed during the current study.

## Abbreviations

AD: Academic Detailing
AUD: Alcohol Use Disorder
CARE-VASH: Coordinated Access for Addiction Recovery and Equity in VA Supportive Housing
CBT: Cognitive Behavioral Therapy
CBT-SUD: Cognitive Behavioral Therapy for Substance Use Disorders
CE: Consumer Engagement
DUA: Data Use Agreement
EBP: Evidence-Based Practice
ED: Emergency Department
EHR: Electronic Health Record
FTE: Full-Time Equivalent
HEIF: Health Equity Implementation Framework
HEV: Homeless-Experienced Veteran
HUD-VASH: Department of Housing and Urban Development VA Supportive Housing
IIW: Inverse Intensity Weighing
MAT: Medication for Addiction Treatment
MAUD: Medications for Alcohol Use Disorder
MCA: Managerial Cost Accounting
MOUD: Medications for Opioid Use Disorder
NLP: Natural Language Processing
NNT: Number Needed to Treat
OUD: Opioid Use Disorder
QUERI: Quality Enhancement Research Initiative
RCT: Randomized Controlled Trial
REP: Replicating Effective Programs
REP + CE: Replicating Effective Programs enhanced with Consumer Engagement
RR: Risk Ratio
SHELTER: Scientific Approaches to Improving Housing and Economic Security Outcomes Advanced by a Learning Health System through Data driven Evaluation and Research
SUD: Substance Use Disorder
TA: Technical Assistance
VA: Department of Veterans Affairs
WMD: Weighted Mean Difference
